# *Winogradskyella bathintestinalis* sp. nov., isolated from the intestine of the deep-sea loosejaw dragonfish, *Malacosteus niger*

**DOI:** 10.1099/ijsem.0.006135

**Published:** 2023-10-01

**Authors:** Shona Uniacke-Lowe, Crystal N. Johnson, Catherine Stanton, Colin Hill, Paul Ross

**Affiliations:** 1 School of Microbiology, University College Cork, Cork, Ireland; 2 APC Microbiome Ireland, Cork, Ireland; 3 Teagasc Food Research Centre, Fermoy, Ireland; 4 Department of Biochemistry & Microbiology, Oklahoma State University – Center for Health Sciences, Tulsa, Oklahoma, USA

**Keywords:** deep sea, fish isolate, gut microbiome, sp. nov., *Winogradskyella*

## Abstract

A novel bacterial strain, APC 3343^T^, was isolated from the intestine of a deep-sea loosejaw dragon fish, *Malacosteus niger*, caught at a depth of 1000 m in the Northwest Atlantic Ocean. Cells were aerobic, rod-shaped, yellow/orange-pigmented, non-motile and Gram-negative. Growth of strain APC 3343^T^ was observed at 4–30°C (optimum, 21–25°C), pH 5.5–10 (optimum, pH 7–8) and 0.5–8 % (w/v) NaCl (optimum, 2–4 %). Phylogenetic analysis based on 16S rRNA gene sequences showed that strain APC 3343^T^ was most closely related to members of the genus *Winogradskyella*, with the most closely related type strains being *Winogradskyella algae* Kr9-9^T^ (98.46 % identity), *Winogradskyella damuponensis* F081-2^T^ (98.07 %), *Winogradskyella eximia* CECT 7946^T^ (97.93 %), *Winogradskyella litoriviva* KMM 6491^T^ (97.79 %) and *Winogradskyella endarachnes* HL2-2^T^ (97.79 %). Major fatty acids (>10 % of total) were iso-C_16:0_ 3-OH, iso-C_15:0_, anteiso-C_15:0_ and iso-C_17:0_ 3-OH. The predominant respiratory quinone was menaquinone-6 (MK-6). Polar lipids were phosphatidylethanolamine, three unknown aminolipids and eight unknown lipids. The draft genome sequence was 3.8 Mb in length with a G+C content of 33.43 mol %. Based on the phenotypic characteristics and phylogenetic analysis, strain APC 3343^T^ is deemed to be a novel species of the genus *Winogradskyella*, and for which the name *Winogradskyella bathintestinalis* sp. nov. is proposed. The type strain of this species is APC 3343^T^ (=DSM 115832^T^=NCIMB 15464^T^).

## Introduction

The genus *Winogradskyella* was first proposed by Nedashkovskaya *et al*. [1] as a novel member of the family *Flavobacteriaceae* and has subsequently been amended several times [2–4]. Members of the genus *Winogradskyella* are Gram-stain-negative and produce non-diffusible yellow or orange pigments. They are usually aerobic rod-shaped cells, but facultative anaerobic and coccoid cells have also been found [5, 6]. To date, there are 46 type strains within the genus *Winogradskyella* (https://lpsn.dsmz.de/genus/winogradskyella).

*Winogradskyella* species have been isolated from a wide variety of marine habitats such as seawater [7] and sediment [8], mostly in association with algae [1, 4, 9, 10] and marine invertebrates [2, 11, 12]. Culturable strains have also been recovered from the gills of marine fish [13]. Most recently, 16S rRNA gene amplicons belonging to members of the genus *Winogradskyella* have been detected in human faecal samples, yet no culturable representatives have been isolated [14]. Furthermore, *Winogradskyella* are of increasing biotechnological interest due to their antibiofilm activity [15, 16], exopolysaccharide production [17], heavy metal resistance [17] and bioremediation potential [18, 19].

There are suggestions that some *Winogradskyella* species are extremophilic: many characterized isolates are psychrotrophic, capable of growth below 10°C [7, 20] and some strains have been shown to be resistant to freeze–thaw cycles [17]. *Winogradskyella* gene sequences have been detected in habitats where sub-zero temperatures were recorded [21]; however, growth of *Winogradskyella* isolates at sub-zero temperatures (under laboratory conditions) has not yet been reported. To date, *Winogradskyella ouciana* represents the only type strain of this genus to be isolated from a habitat of extreme hydrostatic pressure (seawater from 7500 m deep) [22]. This is significant in that hydrostatic pressure increases by one atmosphere for every 10 m increase in water depth in the ocean [23].

A *Winogradskyella* strain, designated strain APC 3343^T^, was previously isolated from the intestine of a deep-sea dragon fish, *Malacosteus niger*, by our lab group during a study of the antimicrobial potential of deep-sea fish microbiome isolates [24]. *Malacosteus niger* (a.k.a. Stoplight loosejaw fish) are true deep-sea dwellers and are rarely found above the mesopelagic zone (500 m deep) [25]. They have specific adaptations to survive in their natural habitat such as unusual visual morphology for detecting specific bioluminescent wavelengths in dark waters [26], and a diet that consists predominantly of zooplankton (specifically copepods) but they are also capable of preying on large fish [27]. It has been reported that Flavobacteriales form part of the core gut microbiome of copepods from subtropical Atlantic and Antarctic waters [28, 29] and may accumulate in the copepod gut through feeding on phytoplankton, on which *Flavobacteriaceae* are abundant [29]. This perhaps also suggests a dietary route for strain APC 3343^T^ to the deep-sea fish gut. Furthermore, *Winogradskyella* genomic DNA has been identified in faecal metagenome samples from Antarctic copepods [30]. However, it is yet to be determined if *Winogradskyella* species are core members of the gut microbiome of deep-sea fish.

In this study, we report the taxonomic characterization of strain APC 3343^T^, a novel member of the genus *Winogradskyella*.

## Methods

### Isolate information

Strain APC 3343^T^ was previously isolated from the intestinal tract of a *Malacosteus niger* fish specimen. The fish was collected by research vessels in surveys in international waters near the Grand Banks of Newfoundland in the north-western Atlantic Ocean (43.282 N 49.121 W) from a depth of approximately 1000 m [31]. Bacterial isolates were recovered from fish intestinal samples, as previously reported [24]. In brief, swabs of the intestinal tract were streaked onto Difco marine agar 2216 (MA) and incubated aerobically for 3 weeks at 4°C. A yellow/orange-pigmented colony was selected and purified through sub-culturing on MA. The strain was deposited into the APC Culture Collection (APC Microbiome Ireland, Teagasc Food Research Centre, Moorepark, Fermoy, Co. Cork, Ireland) and designated strain APC 3343^T^. Cells were preserved in 35% (v/v) glycerol suspensions at –80°C. Strain APC 3343^T^ was routinely cultured on MA at 20–25°C.

*Winogradskyella* sp. APC 3343^T^ has been deposited into the National Collection of Industrial, Food and Marine Bacteria (=NCIMB 15464^T^) and the DSMZ-German Collection of Microorganisms and Cell Cultures GmbH (=DSM 115832^T^).

### Genome sequencing, assembly and annotation

Strain APC 3343^T^ was cultured in Difco marine broth 2216 (MB) for 72 h at 20°C. Genomic DNA (gDNA) extraction, whole-genome sequencing and annotation was carried out as previously described [24]. In brief, the bacterial gDNA was extracted using the GeneJET Genomic DNA Purification Kit (Thermo Scientific). The gDNA was sequenced and assembled by MicrobesNG (https://microbesng.com/, University of Birmingham, UK) using the Illumina platform and SPAdes *de novo* assembly method, and assembly quality was checked using quast. The assembled contigs were submitted to GenBank and annotated upon submission using NCBI’s Prokaryotic Annotation Pipeline. Additionally, gene annotations were made with rast and subsystem features determined using seed [32–34]. The presence of secondary metabolite and bacteriocin biosynthetic gene clusters (BGCs) was queried using antiSMASH and bagel4, respectively, as previously reported [24].

### 16S rRNA gene phylogeny

The complete 16S rRNA gene sequence (1522 bp) was extracted from the whole genome sequence data using barrnap (version 0.9, https://github.com/tseemann/barrnap). The 16S sequence was queried using the EzTaxon-e server in the EzBioCloud service [35]. fasta files of the hit sequences were downloaded and imported into mega X [36]. Multiple alignments with the query sequence were created using muscle [37] within mega X. Gaps from the 5′ and 3′ ends were removed manually, and the partial gap deletion (95% site coverage) parameter was applied. Phylogenetic trees (neighbour-joining, maximum-likelihood and maximum-parsimony) were created in mega X and inferred using the Kimura two-parameter method [38]. Tree robustness was guaranteed by bootstrap analyses based on 1000 replicates [39]. The tree was rooted by including a sequence from the family *Flavobacteriaceae* as an outlier; namely, the 16S rRNA gene sequence from *Marixanthomonas spongiae* Hn-E44 (NR 179850.1).

### Genome phylogeny

For the following pairwise average nucleotide identity (ANI) and roary analyses, all available *Winogradskyella* reference genome assemblies (including type strains, *n*=31) were downloaded from the GenBank database (www.ncbi.nlm.nih.gov/data-hub/genome/). Prior to analysis using roary, the assemblies were annotated using prokka [40] to obtain the GFF3 files. The genomes of *W. algae* and *W. damuponensis* have not yet been included in the Type (Strain) Genome Server (TYGS) database nor in GenBank, and we were not able to acquire these type strains for this study.

ANI values were calculated between APC 3343^T^ and the *Winogradskyella* reference genome assemblies. Pairwise ANI values were calculated with Pyani (version 0.2.12 [41]) using the ANIm method [42]. The DSMZ TYGS annotation platform (https://tygs.dsmz.de/) [43] was used to calculate digital DNA–DNA hybridization (dDDH) values between APC 3343^T^ and related type strains from the database. roary [44] was used to determine and create alignments of the core genes of APC 3343^T^ and the *Winogradskyella* reference genomes. The genomic assembly from *Marixanthomonas spongiae* Hn-E44 (GCA003095375.1) was also annotated and included as an outlier (roary parameters: 90% blast ID, 99% of isolates that must have gene, 100000 cluster limit). RaxML [45] was used to generate a maximum-likelihood phylogenetic tree from the core gene alignment, which was then visualized in mega X.

### Biochemical and phenotypic characterization

The following tests were performed on strain APC 3343^T^ and, unless stated otherwise, were carried out at 25°C under aerobic conditions. Growth at different temperatures was tested at 4, 9, 21, 25, 30, 37, 40 and 44°C on MA. NaCl range for growth was tested by measuring the optical density at OD_600nm_ in MB with the NaCl concentration adjusted within the range 0–16% w/v (in increments of 1% from 0–10% NaCl, and in increments of 2% from 12–16% NaCl). The pH range for growth was tested in MB from pH 5 to 11 (in increments of 0.5 from pH 5–9 and increments of 1 from pH 9–11). The pH was adjusted by the addition of 1 M HCl and/or 1 M NaOH.

Anaerobic growth was assessed on MA incubated in an AnaeroGen anaerobic system (Thermo Scientific Oxoid) for up to 14 days at 21°C. Colony morphology was assessed after incubating on MA at 25°C for 3 days. Cells were examined using phase contrast microscopy and scanning electron microscopy (SEM). SEM imaging was carried out by UCD Conway Imaging Core (UCD Conway Institute of Biomolecular and Biomedical Research, University College Dublin, Ireland). Gram staining was carried out according to standard methods. Production of oxidase was tested using oxidase strips (Millipore) according to manufacturer’s instructions. Production of catalase was tested by transferring a mass of colonies onto a glass slide and exposing them to 1–2 drops of 3% hydrogen peroxide and observing for effervescence. Observation for motility was carried out using the hanging drop method according to the method outlined by Bernardet *et al*. [46] except that a cavity microscope slide was used. The presence of flexirubin-type pigments was tested using the KOH method as described by Bernardet *et al*. [46].

Artificial seawater (ASW) salts base used in the subsequent tests was prepared, as per Kurilenko *et al*. [9] and Bruns *et al*. [47], as follows: 30 g l^−1^ NaCl, 5.94 g l^−1^ MgSO_4_·7H_2_O, 4.53 g l^−1^ MgCl_2_·6H_2_O, 0.64 g l^−1^ KCl and 1.3 g l^−1^ CaCl_2_·2H_2_O. Hydrolysis of DNA was determined by plating on DNase Test Agar (Thermo Scientific) supplemented with ASW salts; following incubation, the DNase plates were flooded with 1M HCl and observed for zones of clearance around colonies. Hydrolysis of casein was tested on agar-medium (1.5% agar) containing ASW salts supplemented with 10% skimmed milk powder. Hydrolysis of cellulose was tested on agar medium containing ASW salts supplemented with 2 g l^−1^ cellulose and 5 g l^−1^ peptone; following incubation, the plate was then stained with 1% Congo red, washed with 1 M NaCl and observed for zones of clearance around colonies. Assimilation of Tween 80 and starch was tested by plating on ASW-based medium containing ASW salts, 5 g l^−1^ peptone, 1g l^−1^ yeast extract, 0.1 g l^−1^ K_2_HPO_4_, 15 g l^−1^ agar and 1% v/v Tween 80 or 0.2% w/v starch. Production of hydrogen sulphide was determined using Watman lead acetate strips (Sigma-Aldrich) suspended above an inoculum of the strain in MB for up to 7 days.

Remaining biochemical tests were carried out using the API 20E and API 20NE kits (bioMérieux) incubated at 25°C for up to 5 days, the API ZYM kits, incubated at 25°C for 18 h; and the GEN III MicroPlate (Biolog) incubated at 25°C for up to 7 days. Results were recorded every day. Bacterial suspensions were prepared in ASW (3% NaCl) for the API test kits. For the GEN III MicroPlate, protocol B was used with inoculating fluid B (IFB) supplemented with 2% NaCl. Due to the mucoid nature of strain APC 3343^T^, the inoculum for the IFB was prepared from broth culture. In brief, an overnight culture of strain 3343^T^ was resuspended and washed in PBS. The PBS cell suspension was then used to inoculate the IFB to the correct cell density.

Analyses of cellular fatty acids, polar lipids and respiratory quinones were carried out by DSMZ (Leibniz Institute DSMZ-German Collection of Microorganisms and Cell Cultures GmbH, Braunschweig, Germany). For fatty acid and polar lipid analyses, strain APC 3343^T^ was cultivated in MA at 25°C. Cellular fatty acids were determined using the Sherlock MIS (midi) system (version 6.1, TSBA40 method, TSBA6 calculation).

Susceptibility to antibiotics was determined using the disc-diffusion method on MA with commercial antibiotics discs (Oxoid) with the following antibiotics: ampicillin (10 μg), chloramphenicol (30 μg) erythromycin (15 μg), gentamicin (10 μg), kanamycin (30 μg), lincomycin (15 μg), neomycin (30 μg), novobiocin (5 μg), oleandomycin (15 μg), penicillin G (10 U), polymyxin B (300 U), rifampcin (30 μg), streptomycin (10 μg) and tetracycline (30 μg). Zone diameters were measured after 36 h at 25°C (aerobic). Due to the lack of CLSI guidelines for interpretation criteria for the genus *Winogradskyella*, antibiotic susceptibility and resistance was interpreted as no growth or growth, respectively, in accordance with previous species descriptions of taxonomic neighbours [9, 20].

## Results

### Genomic features and characterization

**Fig. 1:**
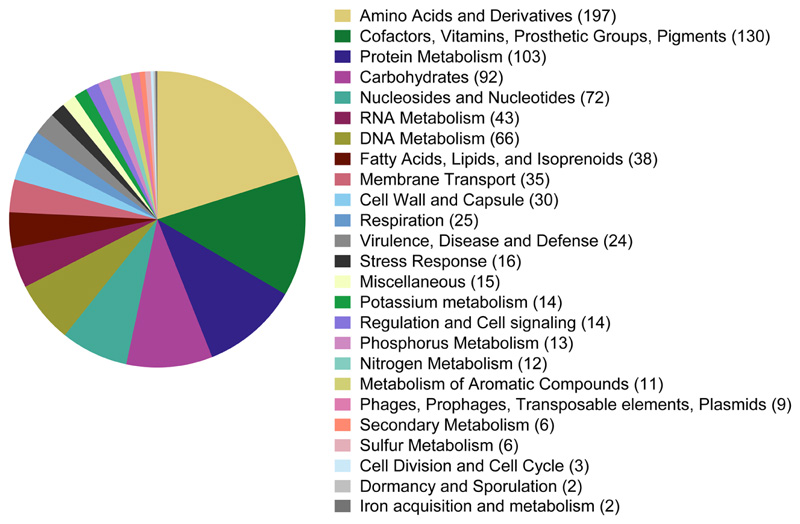
Overview of rast annotation of subsystem features from the draft genome of APC 3343^T^. The counts of features per subsystem category are given. The total number of subsystem features was 978.

The draft genome assembly of strain APC 3343^T^ was 3832957 bp long with a G+C content of 33.43%. The draft assembly consisted of 53 contigs. An overview of the general characteristics of the draft genome of strain APC 3343^T^ is given in Table 1. An overview of the rast annotation of subsytem features is given in Fig. 1. In total, 978 subsytem features were identified across 25 categories. The largest subsystem category was ‘amino acids and derivatives’ for which 197 features were identified, representing 20% of the overall features. The second largest category was ‘cofactors, vitamins, prosthetic groups and pigments’ for which 130 (13%) features were identified; this category included genes associated with riboflavin metabolism and Vitamin B biosynthesis. Some other interesting subsystems in this strain identified by rast included heavy metal resistance (copper homeostasis, five features; cobalt–zinc–cadmium resistance, four; resistance to fluoroquinolones, two) and stress response (including cold shock, one; oxidative stress, nine). Among the secondary metabolism features were genes associated with plant alkaloid synthesis and auxin biosynthesis. No features associated with motility were identified, which is in agreement with the phenotypic motility test results. It was also noted that more features were dedicated to protein metabolism (103) than carbohydrate metabolism (92).

### Secondary metabolite BGC and bacteriocin gene screening

Four secondary metabolite BGCs were identified using antiSMASH: two terpenes, one type-3 polyketide synthase (T3PKS) and one hybrid non-ribosomal peptide synthetase (NRPS) – type-1 polyketide synthase (T1PKS). One of the terpene BGCs was most similar to that of carotenoid, at 28% similarity. The T3PKS shared 8% similairity to a beta-lactam BGC in the database from an uncultured organism. The remaining BGCs of APC 3343^T^ had no identifiable cluster hit within the antiSMASH database. bagel4 identified one sactipeptide BGC, though it lacked a core sactipeptide gene. One of the genes in the cluster shared 41.4% similarity to *moaA*, a gene encoding the radical S-adenosyl-L-methionine (SAM) family protein GTP-3′,8-cyclase (Uniprot B7ULX8). An overview of the antiSMASH and bagel4 screening results is given in Table S1.

### 16S rRNA phylogenetic analysis

**Fig. 2:**
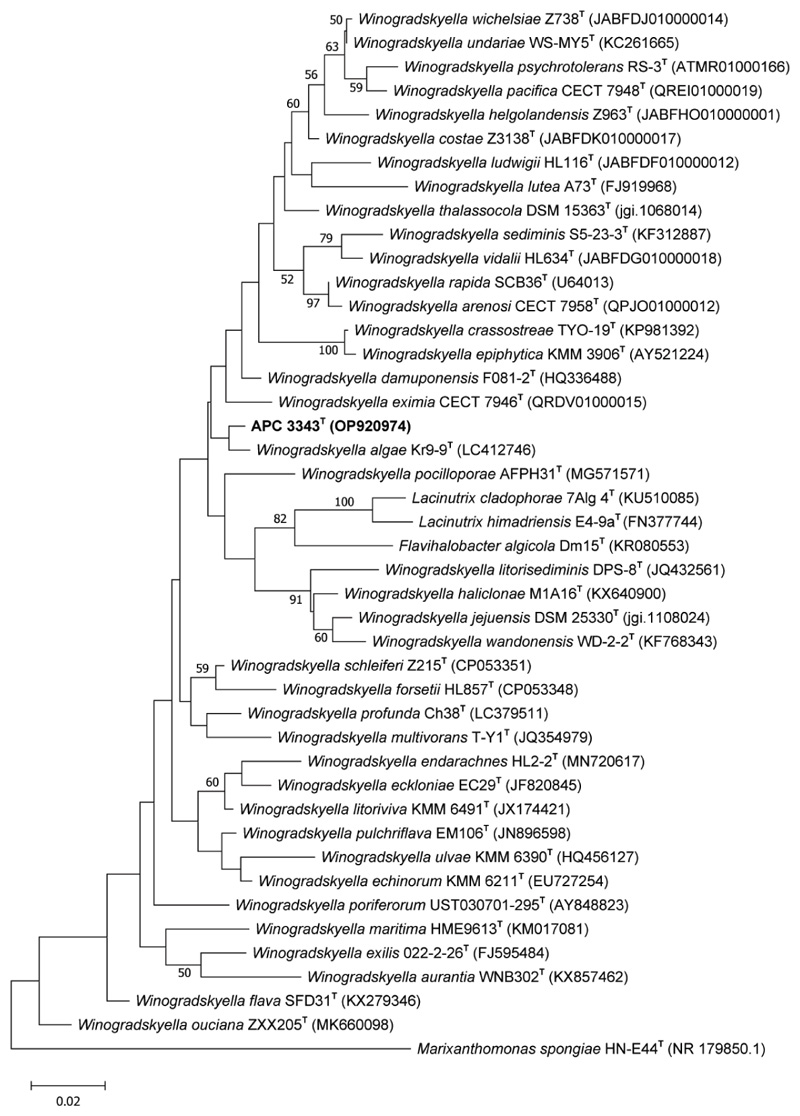
Maximum-likelihood tree based on 16S rRNA gene sequences showing the relationship of strain ACP 3343^T^ and other members of the genus *Winogradskyella* and related taxa. Distances were calculated based on the Kimura two-parameter model. Bootstrap values as% based on 1000 replications (>50%) are shown beside each branch node. Bar, 0·01 substitution per nucleotide position.

The 16S rRNA gene sequence similarity analysis indicated that APC 3343^T^ was most closely related to members of the genus *Winogradskyella* within the family *Flavobacteriaceae*. The APC 3343^T^ sequence showed the highest similarity to that of *Winogradskyella algae* Kr9-9^T^ [9] (98.46% similarity), followed by *Winogradskyella damuponensis* F081-2^T^ [20] (98.07%), *Winogradskyella eximia* CECT 7946^T^ (=KMM 3944^T^) [1] (97.93%), *Winogradskyella litoriviva* KMM 6491^T^ [7] (97.79%) and *Winogradskyella endarachnes* HL2-2^T^ [10] (97.79%). These closely related strains were selected to be used as reference strains for the comparative phenotypic, biochemical and chemotaxonomic analyses. The 16S rRNA sequence similarity values between APC 3343^T^ and the reference strains are below the currently accepted threshold of 98.7% for species [48], indicating that strain 3343^T^ represents a separate species of the genus *Winogradskyella*. In the maximum-likelihood (Fig. 2) and neighbour-joining (Fig. S1, available in the online version of this article) phylogenetic trees, generated from the 16S rRNA gene sequence alignments, APC 3343^T^ placed closely to *Winogradskyella algae* Kr9-9^T^. In the maximum-parsimony phylogenetic tree APC 3343^T^ formed its own clade (Fig. S2).

### Genome phylogenetic analysis

**Fig. 3:**
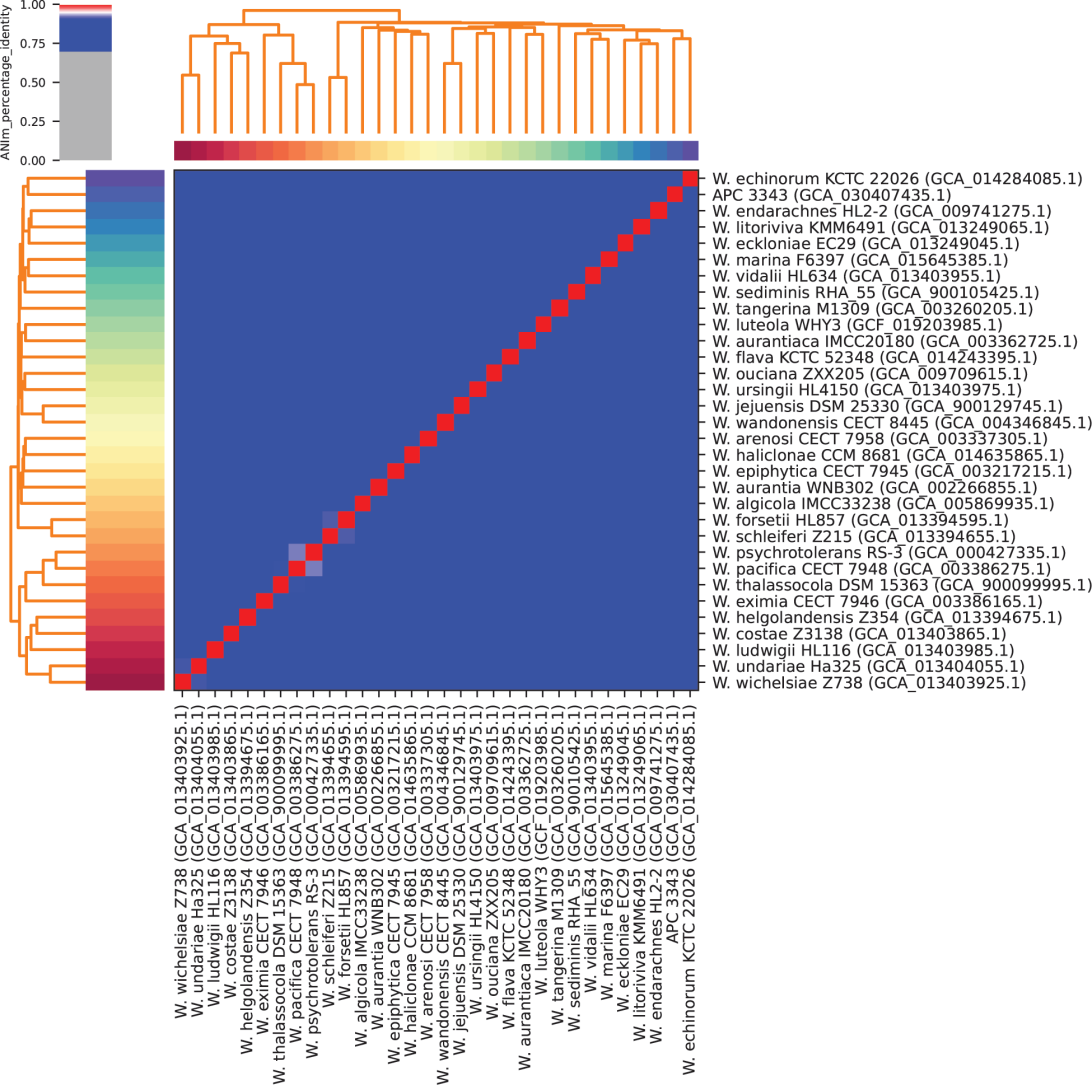
Pairwise average nucleotide identity (ANI) of APC 3343^T^ and all available *Winogradskyella* type strain reference genomes from the GenBank database. ANI values were calculated using Pyani using the ANIm method. Blue cells correspond to an ANI of <95%, indicating separate species. Red cells correspond to an ANI of >95%, indicating the same species. The colour intensities lighten as the ANI value approaches 95%. The dendrograms are created by hierarchal clustering of the pairwise ANI values.

The ANI values between APC 3343^T^ and the available *Winogradskyella* type strain reference genomes ranged from 83.69 to 85.03% (Fig. 3). The ANI alignment coverage ranged from 1 to 28% (data not shown). An overview of the dDDH values and G+C content difference between APC 3343^T^ and the top TYGS database hits are given in Table S2. The dDDH (d4) values between APC 3343^T^ and *W. eximia*, *W. litoriviva* and *W. endarachnes* were 23.4, 22.6 and 21.5%, respectively. ANI and dDDH values of 95–96 and 70%, respectively, are considered the general cut-off for species and should be assessed as part of the overall genome related index when the 16S rRNA gene sequence identity values are ≥98.7% [48]. The ANI and dDDH values in this study indicate that strain APC 3343^T^ represents a separate species among these *Winogradskyella* reference genomes. roary was used also to assess phylogeny based on the alignment of 33 core genes from strain APC 3343^T^, the reference *Winogradskyella* genomes and the outlier, *Marixanthomonas spongiae* Hn-E44. The maximum-likelihood phylogenetic tree generated from this core gene alignment is shown in Fig. S3.

### Biochemical and phenotypic characterization

**Fig. 4:**
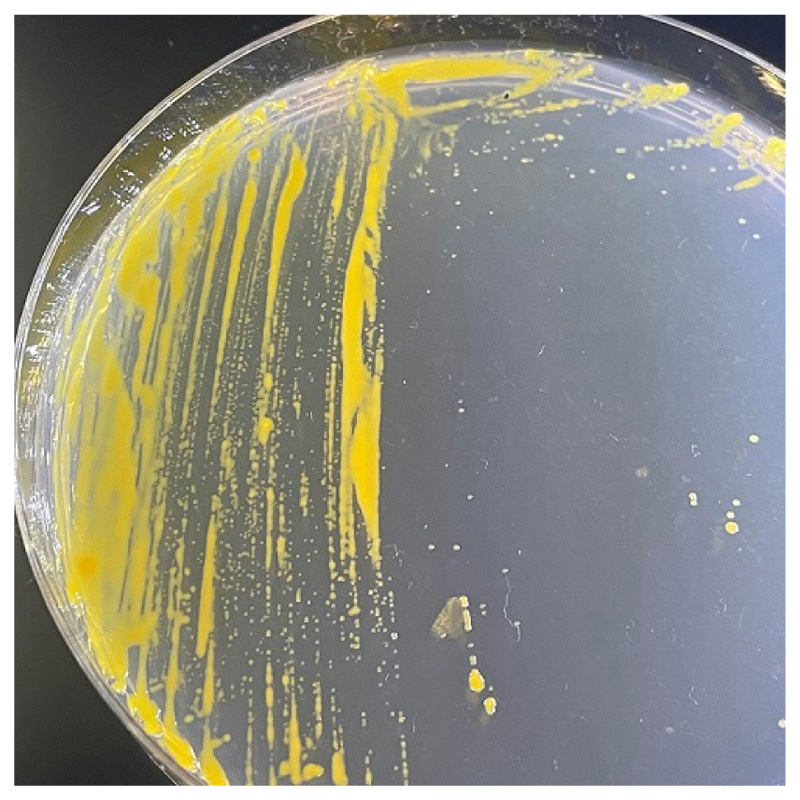
Colonies of strain APC 3343^T^ (after 3 days on MA at 25°C).

**Fig. 5:**
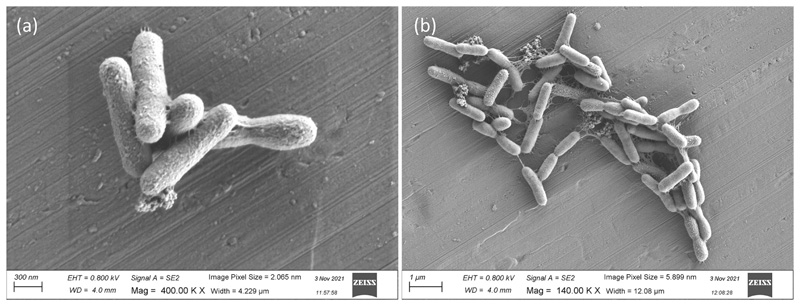
Scanning electron microscopy image of APC 3343^T^ at ×400K (a) and ×140K (b).

Strain APC 3343^T^ was found to be aerobic, Gram-stain-negative and non-motile. Colonies were circular, smooth and convex, measuring 0.8–1.2 mm after incubating on MA at 25°C for 3 days (Fig. 4). Colonies produced a pale yellow, non-flexirubin-type pigment and intensified in colour with prolonged incubation time, resulting in colonies turning progressively orange in colour. Cells were rod-shaped (1–1.5 μm x 0.3 μm) without flagella (Fig. 5). SEM images of clustering cells revealed the presence of network-like structures on the cell surface of strain APC 3343^T^ (Fig. 5b). Similar structures have been described for *W. thalassocola* KMM 3907^T^, *W. epiphytica* KMM 3906^T^ and *W. eximia* KMM 3944^T^, but are not present on other *Winogradskyella* species such as *W. haliclonae* [49]. Such structures are believed to be an adaptation for cellular attachment and aggregate formation [1].

Differential characteristics between APC 3343^T^ and the reference *Winogradskyella* strains (*Winogradskyella algae* Kr9-9^T^ [9], *Winogradskyella damuponensis* F081-2^T^ [20], *Winogradskyella eximia* KMM 3944^T^ [1, 4], *Winogradskyella litoriviva* KMM 6491^T^ [7] and *Winogradskyella endarachnes* HL2-2^T^ [10]) are provided in Table 2. All data for strain APC 3343^T^ is from this study. The phenotypic and biochemical data for the reference strains was acquired from the respective literature. In the phenotypic assays, APC 3343^T^ shared a number of characteristics that are in accordance with *Winogradskyella* species [1], such as a requirement for NaCl, alkaline phosphatase, oxidase and catalase and gelatin degradation activities, except strain APC 3343^T^ did not degrade DNA. The temperature range for growth of strain APC 3343^T^, of 4–30°C (optimum 21–25°C), was lower than those of the reference *Winogradskyella* strains [1, 4, 7, 8, 11, 20]. The NaCl range for growth of strain APC 3343^T^, at 0.5–8% (optimum, 2–4%), was wider than those of the reference strains, though almost identical to that of *W. litoriviva* KMM 6491^T^ (0.5–7% NaCl) [7]. The pH range for growth of strain APC 3343^T^ was pH 5.5–10 (optimum, pH 7–8), equal to that of *W. algae* Kr9-9^T^ [9] and *W. litorviva* KMM 6491^T^ [7]. The main differences observed between strain APC 3343^T^ and the reference strains in the phenotypic assays were in its ability to produce, albeit weakly, lipase and *β*-glucosidase. Strain APC 3343^T^ also uniquely showed some *β*-galactosidase activity with paranitrophenyl-*β*-d-galactopyranoside as the substrate (API 20NE PNPG assay).

It is to be noted that the Biolog GEN III plate results were difficult to interpret due to apparent weak metabolic activity by strain APC 3343^T^. This is possibly an indication of the fastidious nature of this strain or an inability to grow on a single carbon source. A second assay using the GEN III MicroPlates and protocol C2 with inoculating fluid C (for fastidious organisms) was also carried out; however, some colour production (oxidation) was observed in the negative control well and therefore these results were not used. From the API ZYM assay results, APC 3343^T^ was positive for alkaline phosphatase, esterase, esterase lipase, leucine arylamidase, valine arylamidase, cystine arylamidase, acid phosphatase and naphthol-AS-BI-phosphohydrolase activity, weakly positive for lipase (C14), α-glucosidase and *β*-glucosidase activity, and negative for trypsin, α-chymotrypsin, α-galactosidase, *β*-galactosidase (2-naphthyl-*β*-d-galactopyranoside as substrate), *β*-glucuronidase, *N*-acetyl-*β*-glucosaminidase, mannosidase and fucosidase activity.

The predominant (>10% of total) cellular fatty acids of strain APC 3343^T^ were iso-C_16:0_ 3-OH (13.3%), iso-C_15:0_ (13.1%), anteiso-C_15:0_ (11.9%) and iso-C_17:0_ 3-OH (10.6%). The full list of identified cellular fatty acids in this strain and those of the reference strains are provided in Table 3. APC 3343^T^ contained a unique fatty acid profile compared to the reference strains, particularly with regard to the hydroxy and saturated fatty acids and summed features [4, 7, 8, 11, 20]. Unusually, a trace amount (<1%) of an unknown fatty acid of an estimated carbon length of 13.6 was identified in strain APC 3343^T^. The sole respiratory quinone was menaquinone 6 (MK6), in accordance with members of the genus *Winogradskyella* [1]. The cellular polar lipid profile of APC 3343^T^ consisted of phosphatidylethanolamine, three unknown aminolipids and eight unknown lipids (Fig. S4) and, when compared to the reference strains, was most similar to that of *W. algae* Kr9-9^T^ [9] except that APC 3343^T^ contained a higher number of unknown/unidentified lipids (Table S3).

Strain APC 3343^T^ was susceptible to ampicillin (10 μg), chloramphenicol (30 μg), erythromycin (15 μg), lincomycin (15 μg), novobiocin (5 μg), oleandomycin (15 μg), penicillin G (10 U), rifampcin (30 μg) and tetracycline (30 μg); and resistant to gentamicin (10 μg), kanamycin (30 μg), neomycin (30 μg), polymyxin B (300 U) and streptomycin (10 μg). The antibiotic susceptibility profile of strain 3343^T^, including the zone diameter measurements, is given in Table S4. Antibiotic susceptibility and resistance were interpreted as no growth or growth, respectively, in accordance with previous species descriptions of taxonomic neighbours [9, 20] and due to the lack of CLSI guidelines for interpretation criteria for the genus *Winogradskyella*. It was also noted that members of the *Winogradskyella* genus lack standardized protocols for growth conditions from which phenotypic information has been described, such as the use of MA rather than Mueller–Hinton, and variable growth temperatures among species representatives ranging from 28°C for *W. algae* and 25°C for *W. damuponensis*, rather than the recommended incubation temperature of 18°C for psychrophilic *Flavobacteriaceae* [20]. Only three species within this genus (*W. lutea* [3], *W. flava* [50] and *W. damuponensis* [20]) were reported following CLSI guidelines, with none detailing the break-point values used to assign resistance/susceptibility, and only one (*W. maritima*) stating interpretations were performed according to the CLSI criteria for the family *Enterobacteriaceae* [51]. This divergence in antimicrobial susceptibility testing highlights a deficiency in the standardization within this group, however, we have reported our results in line with the existing literature.

Strain APC 3343^T^ clearly differed from the most closely related strain, *W. algae* Kr9-9^T^ [9], in its inability to reduce nitrate or hydrolyse cellulose, lack of α-chymotrypsin activity and its sensitivity to ampicillin, erythromycin, lincomycin and tetracycline (as well as in the above-mentioned range for growth parameters). These assays may be useful in differentiating these two strains.

Based on the combination of phylogenetic distance, phenotypic and biochemical characteristics, strain APC 3343^T^ represents a novel species for which we propose the name *Winogradskyella bathintestinalis* sp. nov.

## Description of *Winogradskyella Bathintestinalis* Sp. Nov

*Winogradskyella bathintestinalis* (bath.in.tes.ti.na’lis. Gr. masc. adj. *bathys*, deep; N.L. masc. adj. *intestinalis*, pertaining to the intestine; N.L. fem. adj. *bathintestinalis*, of the deep intestine).

Cells are Gram-stain-negative, rod-shaped (1–1.5×0.3 μm), aerobic, non-motile and non-flagellated. Colonies are pale yellow/orange-pigmented, raised, circular, shiny/mucoid with smooth margins after incubation on MA at 25°C for 3 days. Cell-associated filaments are produced. Pigments are non-flexirubin-type. Growth occurs at 4–30°C (optimum, 21–25°C), 0.5–8% (w/v) NaCl (optimum, 2–4%) and pH 5.5–10 (optimum, pH 7–8). Positive for oxidase and catalase activity. H_2_S is weakly produced under aerobic conditions. Positive for hydrolysis of Tween 80, weakly positive for hydrolysis of starch. Agar, casein, cellulose and DNA are not hydrolysed. From the API 20E assay results: positive for gelatinase and weakly positive for tryptophan deaminase activity; negative for activity of *β*-galactosidase (OPNG assay), arginine dihydrolase (anaerobic), lysine decarboxylase (anaerobic), ornithine decarboxylase (anaerobic) and urease (anaerobic); negative for citrate utilization and H_2_S production (anaerobic) and negative for acid production from d-glucose, d-mannitol, inositol, d-sorbitol, l-rhamnose, sucrose, melibiose amygdalin and l-arabinose. From the API 20NE assay results: positive for hydrolysis of gelatin and aesculin; weakly positive for activity of *β*-galactosidase (PNPG assay); negative for nitrate reduction, indole production, fermentation of glucose, arginine dihydrolase activity (anaerobic) and urease production (anaerobic), and negative for assimilation of d-glucose, l-arabinose, d-mannose, d-mannitol, *N*-acetyl-glucosamine, maltose, potassium gluconate, capric acid, adipic acid, malic acid, trisodium citrate and phenylacetic acid. From the Biolog GENIII assay: positive for oxidation of trehalose, sucrose, stachyose, melibiose, *N*-acetyl-*β*-d-mannosamine, *N*-acetyl-d-galactosamine, *N*-acetyl neuraminic acid, l-fucose, l-glutamic acid, acetoacetic acid and acetic acid; weakly positive for oxidation of maltose, cellobiose, gentobiose, turanose, lactose, α-d-glucose, d-mannose, d-fructose, d-galactose, d-fucose, l-rhamnose, sorbitol, d-mannitol, d-arabitol, *myo*-inositol, glycyl-l-proline, glucuronamide, mucic acid and α-keto-glutaric acid; negative for the remaining 40 substrates. The sole cellular respiratory quinone is menaquinone-6 (MK6). The cellular polar lipids are phosphatidylethanolamine, three unknown aminolipids and eight unknown lipids. The major cellular fatty acids are iso-C_16:0_ 3-OH, iso-C_15:0_, anteiso-C_15:0_ and iso-C_17:0_ 3-OH.

The type strain, APC 3343^T^ (=DSM 115832^T^=NCIMB 15464^T^), was isolated from the intestinal tract of a deep-sea fish, *Malacosteus niger*, from a depth of approximately 1000 m in the Northwest Atlantic Ocean (43.282 N 49.121 W). The DNA G+C content of the type strain is 33.43 mol%.

The GenBank accession numbers for the 16S rRNA gene sequence and the genome sequence of strain APC 3343^T^ are OP920974 and JASDDK000000000, respectively.

## Supplementary Material

Supplementary information

## Figures and Tables

**Table 1:** General characteristics of the draft genome of strain APC 3343^T^

Genomic feature	
Draft genome size (bp)	3832957
G+C content (mol%)	33.43
N50	1042040
L50	2
Number of contigs	53
Largest contig	1191771
CDS	3371
RNA genes (rast)	37
rRNA genes (rast)	1
rRNA genes (barrnap)	6
tRNA genes (rast)	36
AMR genes (ABRicate)	0

**Table 2:** Differential characteristics of isolate APC 3343^T^ and closely related *Winogradskyella* type strains Strains: 1, APC 3343^T^ (data from this study); 2, *W. algae* Kr9-9^T^ (data from Kurilenko *et al*. [9]); 3, *W. damuponensis* F081-2^T^ (data from Lee *et al*. [20]); 4, *W. eximia* KMM 3944^T^ (data from Nedashkovskaya *et al*. [1] and Nedashkovskaya *et al*. [4]); 5, *W. litoriviva* KMM 6491^T^ (data from Nedashkovskaya *et al*. [7]); 6, *W. endarachnes* HL2-2^T^ (data from Xu *et al*. [10]). nd, No data available; +, positive; –, negative; w, weakly positive.

Characteristic	1	2	3	4	5	6
Growth at/with:						
37°C	−	−	−	−	−	+
Temperature range (°C)	4–30	7–36	4–35	4–35	4–34	20–40
Temperature optimum (°C)	21–25	28–30	25–30	21–23	25–28	30
NaCl range (%, w/v)	0.5–8	2–6	1–5	1–8	0.5–7	0–5
pH range	5.5–10	5.5–10.0	6.0–9.5	ND	5.5–10	5.5–8
Nitrate reduction	−	+	−	−	−	nd
Glucose fermentation	−	−	−	−	+	+
H_2_S production	w	w	−	−	w	nd
Hydrolysis:						
Aesculin	+	+	+	+	+	nd
Casein	−	−	−	+	−	nd
DNA	−	−	+	−	−	nd
Starch	w	+	−	+	+	nd
Assimilation of:						
Cellobiose	w	nd	+	−	+	+
d-Galactose	+	nd	+	−	+	nd
d-Glucose	−	−	+	+	+	+
d-Mannose	−	−	+	+	+	+
Mannitol	−	−	+	+	−	+
Enzyme activity (API ZYM):						
Esterase	+	+	+	+	w	+
Esterase lipase	+	+	+	+	+	+
Lipase (C14)	w	−	−	−	−	+
Leucine arylamidase	+	+	+	−	+	+
Valine arylamidase	+	+	+	+	+	+
Cystine arylamidase	+	w	+	−	+	+
Trypsin	−	−	−	−	−	+
α-Chymotrypsin	−	+	−	−	+	+
α-Galactosidase	−	−	−	+	−	−
α-Glucosidase	w	−	−	−	+	+
*β*-Glucosidase	w	−	−	−	−	−
Susceptibility to antibiotics:						
Ampicillin	+	−	+	−	+	+
Chloramphenicol	+	+	+	+	+	+
Erythromycin	+	−	+	+	+	+
Gentamicin	−	−	−	+	−	−
Kanamycin	−	−	−	+	−	−
Lincomycin	+	−	+	+	+	+
Neomycin	−	−	−	+	−	−
Leandomycin	+	+	+	−	+	nd
Penicillin G	+	+	+	−	+	nd
Polymyxin B	−	−	−	+	−	−
Rifampcin	+	+	+	+	+	nd
Streptomycin	−	−	−	+	−	−
Tetracycline	+	−	+	nd	+	+

**Table 3:** Cellular fatty acid compositions (%) of APC 3343^T^ and closely related type strain members of the genus *Winogradskyella* Strains: 1, APC 3343^T^ (data from this study); 2, *W. algae* Kr9-9^T^ [9]; 3, *W. damuponensis* F081-2^T^ [20]; 4, *W. eximia* KMM 3944^T^ [1]; 5, *W. litoriviva* KMM 6491^T^ [7]; 6, *W. endarachnes* HL2-2^T^ [10]. Major fatty acids (>10%) are highlighted in bold. Fatty acids amounting <1.0% for all reference strains are not shown. TR, Trace amount (<1%); −, not detected. ECL, estimated carbon length.

Fatty acid	1*	2†	3*	4†	5†	6‡
Saturated:						
C_15:0_	tr	8.3	5.3	7.9	6.4	–
C_16:0_	–	1.8	tr	–	tr	**19.6**
C_17:0_ cyclo	–	–	–	2.3	–	–
C_18:0_	–	–	–	–	–	2.1
Unsaturated:						
C_15:1_	–	2.7	–	–	–	–
C_15:1_ ω6*c*	2.8	–	1.9	–	1.4	–
C_15:1_ ω8*c*	–	–	–	–	1.7	–
C_16:1_	–	3.2	–	–	–	–
C_17:1_ ω6*c*	2.8	–	1.1	–	–	–
C_18:1_ ω5*c*	–	–	2.5	–	–	–
Branched chain:						
iso-C_12:0_	–	–	–	–	–	1.3
ios-C_13:0_	–	–	–	–	–	1.3
ios-C_14:0_	1.4	1.3	1.3	1.4	–	tr
ios-C_15:0_	**13.1**	**14.4**	**25.3**	**25.6**	**19.4**	**37.0**
ios-C_15:1_	–	9.9	–	**11.4**	**15.5**	–
ios-C_15:1_ G	9.0	–	**14.6**	–	–	8.9
ios-C_16:0_	2.2	2.7	1.9	5.7	tr	–
ios-C_16:1_	–	1.8	–	4.7	–	–
ios-C_16:1_ H	4.3	–	2.2	–	–	–
anteiso-C_15:0_	**11.9**	**12.7**	7.8	4.9	9.5	4.1
anteiso-C_15:1_	–	–	–	1.6	4.8	–
anteiso-C_15:1_ A	4.4	3.4	3.9	–	–	–
anteiso-C_17:1_	–	–	–	2.3	–	–
anteiso-C_17:1_ ω9*c*	2.6	–	–	–	–	–
Hydroxy:						
C_15:0_ 2-OH	3.0	–	2.0	1.0	–	tr
C_15:0_ 3-OH	–	–	1.2	–	tr	–
C_16:0_ 3-OH	–	1.5	tr	–	tr	–
C_17:0_ 2-OH	5.2	–	2.6	1.0	–	tr
C_18:0_ 2-OH	–	–	–	–	–	1.0
iso-C_15:0_ 2-OH	–	1.4	–	–	–	1.9
iso-C_15:0_ 3-OH	4.7	3.6	7.6	2.6	9.6	7.8
iso-C_16:0_. 2-OH	–	–	–	–	1.2	–
iso-C_16:0_ 3-OH	**13.3**	**12.9**	6.7	3.2	4.6	1.6
iso-C_17:0_ 3-OH	**10.6**	7.4	9.3	6.7	8.4	8.1
anteiso-C_15:0_ 2-OH	–	1.7	–	–	–	–
anteiso-C_15:0_ 3-OH	–	2.5	–	–	–	–
anteiso-C_17:0_ 3-OH	–	4.5	–	–	4.7	–
Methylated:						
C_16:0_ 10 methyl	–	–	–	6.3	–	–
Other:						
C_16:1_ ω7, iso-C_15:0_ 2-OH	–	–	–	6.1	5.8	–
Unknown	tr (ECL 13.6)	–	–	5.6	–	–
Summed feature 3	7.0 (C_16:1_ ω7*c* / C_16:1_ ω6*c*)	–	2.0 (C_16:1_ ω6*c* and/or C_16:1_ ω7*c*)	–	–	–
Summed feature 9	1.8 (iso-C_17:1_ ω9*c*)	–	2.4 (C_10_-methyl and/or iso-C_17:1_ ω9*c*)	–	–	tr (iso-C_17:1_ ω9*c*/10-methyl-C_16:0_)

* Analysis carried out at 25°C.

† Analysis carried out at 28°C.

‡ Analysis carried out at 30°C.

## Abbreviations

ANI: average nucleotide identity
ASW: artificial seawater
BGC: biosynthetic gene cluster
dDDH: digital DNA–DNA hybridization
IFB: inoculating fluid B
MA: marine agar
MB: marine broth
ONPG: orthonitrophenyl-*β*-d-galactopyranoside
PNPG: paranitrophenyl-*β*-d-galactopyranoside
