# Supplementary material for: Winogradskyella bathintestinalis sp. nov., isolated from the intestine of the deep-sea loosejaw dragonfish, Malacosteus niger
Source: Int J Syst Evol Microbiol. Author manuscript; Available in PMC 2024 Jan 19. (PMC7615552; doi:10.1099/ijsem.0.006135)
Supplement: Supplementary information [file EMS193230-supplement-Supplementary_information.pdf]

Supplementary Materials

Winogradskyella bathintestinalis sp. nov., isolated from the intestine of the deep-sea loosejaw dragonfish, *Malacosteus niger*

Shona Uniacke-Lowe[1,2,3], Crystal N. Johnson[4], Catherine Stanton[2,3], Colin Hill[1,2], Paul Ross[1,2]

[1] Department of Microbiology, University College Cork, Ireland. [2] APC Microbiome Ireland, Cork, Ireland. [3]Teagasc Food Research Centre, Fermoy, Ireland. [4]Department of Biochemistry & Microbiology, Oklahoma State University – Center for Health Sciences, Tulsa, Oklahoma, USA.

* Correspondence: p.ross@ucc.ie

Address: APC Microbiome Ireland, University College Cork, Cork, T12 K8AF, Ireland
Phone: +353 21 490 1322

Table S1: Overview of *in silico* screening for secondary metabolite BGCs and bacteriocin gene clusters using antiSMASH and BAGEL, respectively, within the genome of strain APC 3343^{T}.

Query	Hit type	Node	Most similar known / database hit	% Similarity / match	Accession / motif	Database
APC 3343	Sactipeptides	1	GTP 3',8-cyclase (*moaA*)	41.4	A8MLW5	BAGEL4
	Nrps-T1pks	2	none	-	-	antiSMASH
	T3pks	1	NRP: Betalactam	8	BGC0000350	antiSMASH
	Terpene	4	Carotenoid	28	BGC0000650	antiSMASH
	Terpene	5	none	-	-	antiSMASH

BGC = biosynthetic gene cluster; NRP = non-ribosomal peptide; Nrps = Non-ribosomal peptide synthetase; T1pks = Type one polyketide synthase; T3pks = Type three polyketide synthase.

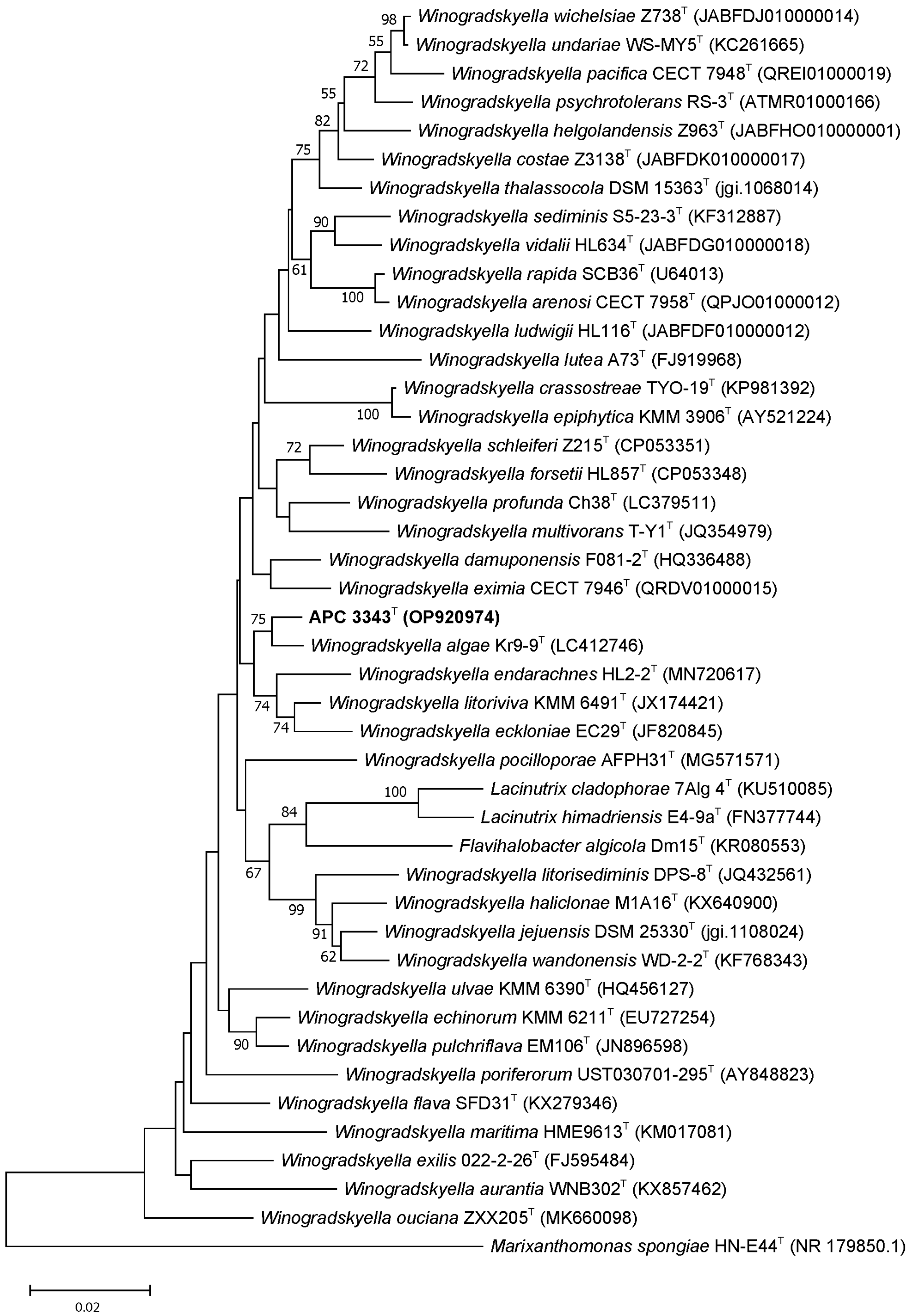

Figure S1: Neighbor-joining phylogenetic tree based on the 16S rRNA gene sequences of APC 3343^T and closely related hits identified through EzBioCloud. Distances were calculated based on the Kimura-2 parameter model and bootstrap analysis of 1000 replicates, values >50% are given at the branching points.

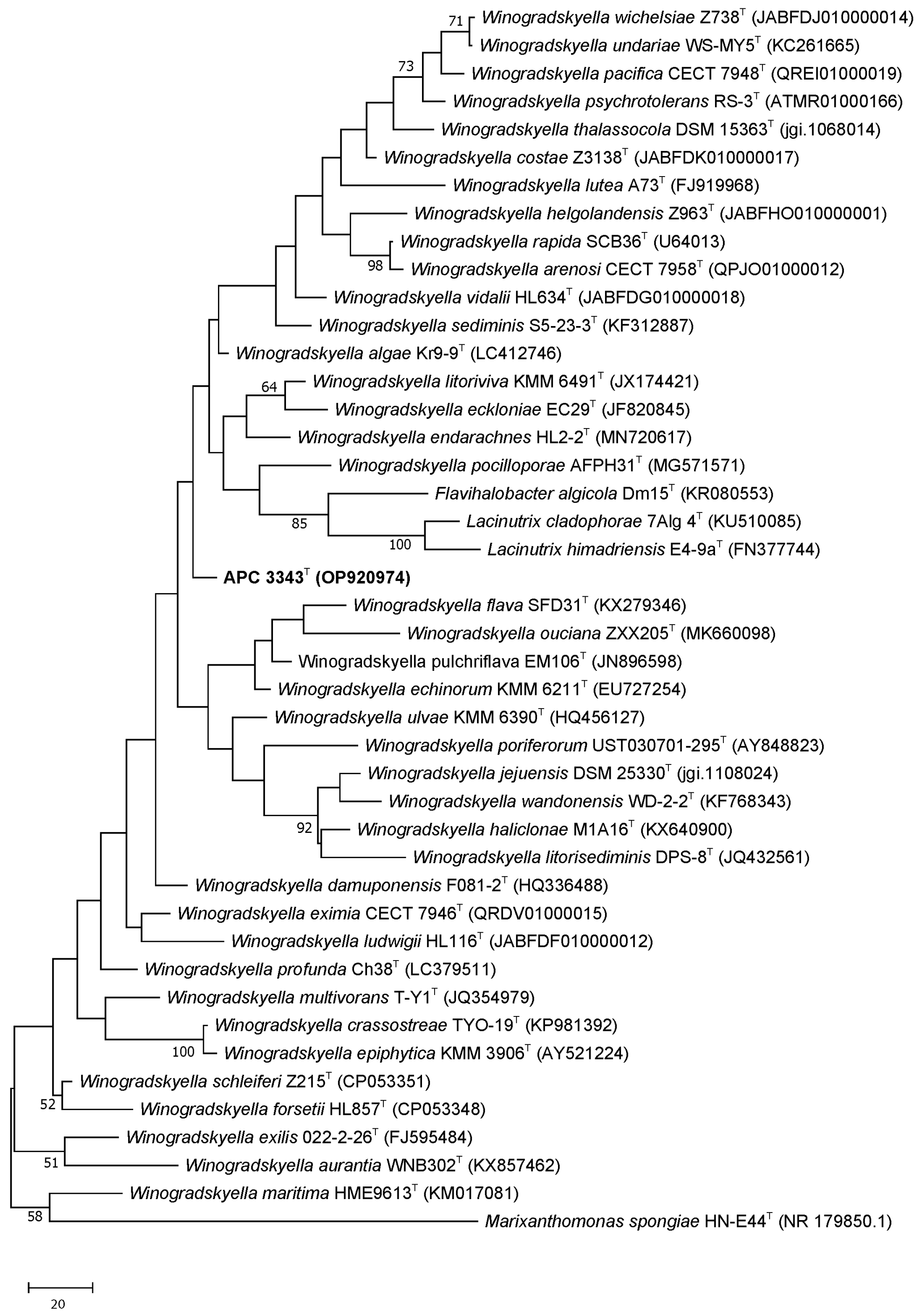

Figure S2: Maximum-parsimony phylogenetic tree based on the 16S rRNA gene sequences of APC 3343^T and closely related hits identified through EzBioCloud. Distances were calculated based on the Kimura-2 parameter model and bootstrap analysis of 1000 replicates, values >50% are given at the branching points.

Table S2: Pairwise dDDH (d4) values and %G+C content differences calculated between APC 3343^T and closely related database hits from the Type Strain Genome Server (TYGS) database. Genome size (bp) of the hit strain is also given.

Query strain	Subject strain	Genome size (bp)	dDDH (d4, in %)	G+C content difference (in %)
**APC 3343**	*Winogradskyella forsetii* HL857	4512651	24.1	1.29
	Winogradskyella schleiferi Z215	4560973	23.8	1.2
	Winogradskyella marina F6397	3974081	23.5	0.16
	Winogradskyella eximia CECT 7946	4235755	23.4	1.09
	Winogradskyella psychrotolerans RS-3	3593001	22.6	0.05
	Winogradskyella litoriviva KMM 6491	3875632	22.6	1.65
	Winogradskyella thalassocola DSM 15363	4571886	22.5	0.15
	Winogradskyella pacifica CECT 7948	4275916	22.3	0.13
	Winogradskyella echinorum KCTC22026(T)	3784537	22.2	1.13
	Winogradskyella echinorum KCTC 22026	3778959	22.2	1.15
	Winogradskyella wichelsiae Z738	3893712	21.5	1.16
	Winogradskyella endarachnes HL2-2	3593001	21.5	1.57
	Winogradskyella eckloniae EC29	3687966	21.3	0.51
	Winogradskyella epiphytica KCTC 12220	3364767	21	0.74
	Winogradskyella arenosi CECT 7958	3666443	20.9	2.24

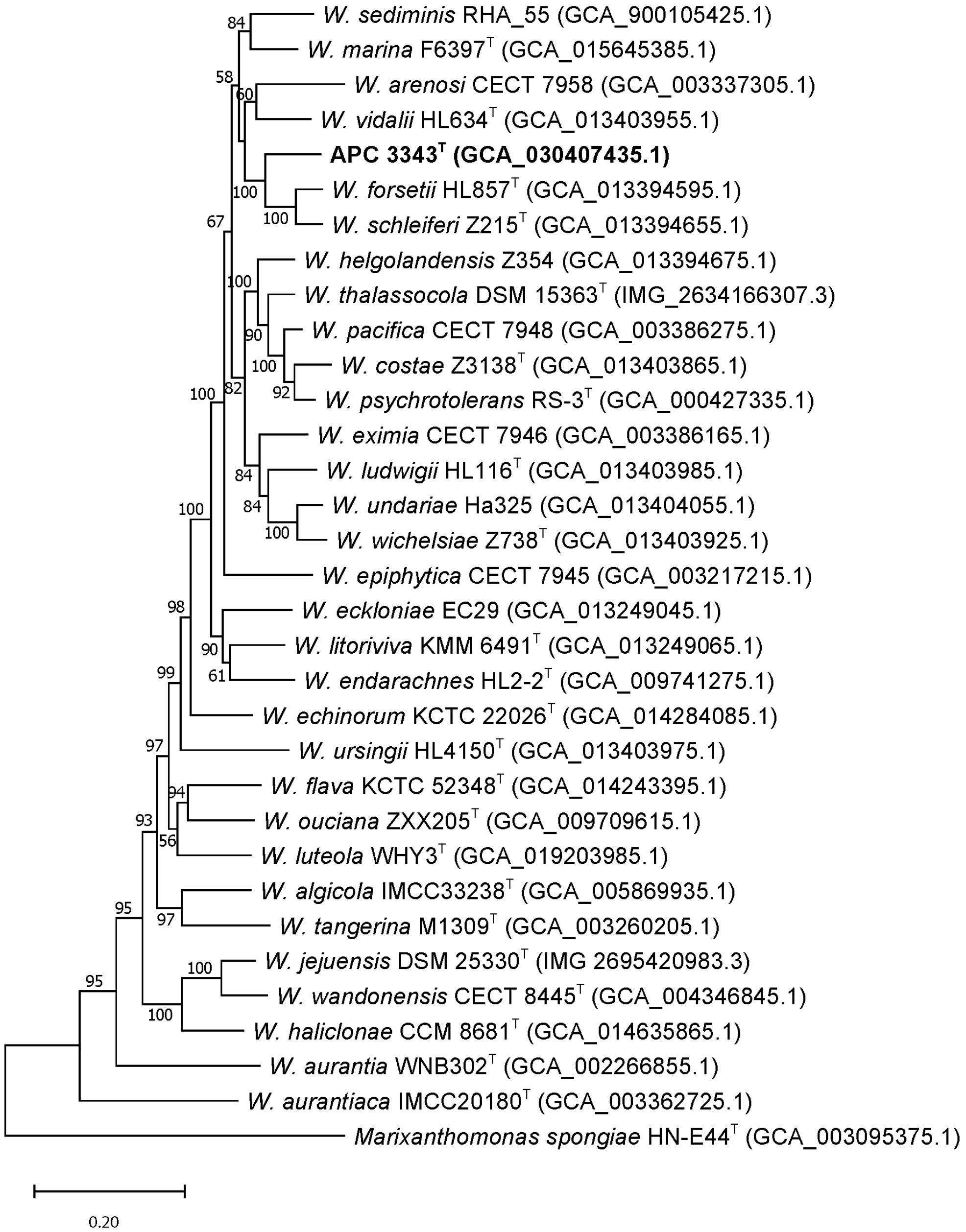

Figure S3: Resulting maximum-likelihood phylogenetic tree based on the alignment of 33 core genes generated by ROARY from strain APC 3343^T and all available *Winogradskyella* spp. reference genomes. *Marixanthomonas spongiae* HN-E44 was included as an outlier.

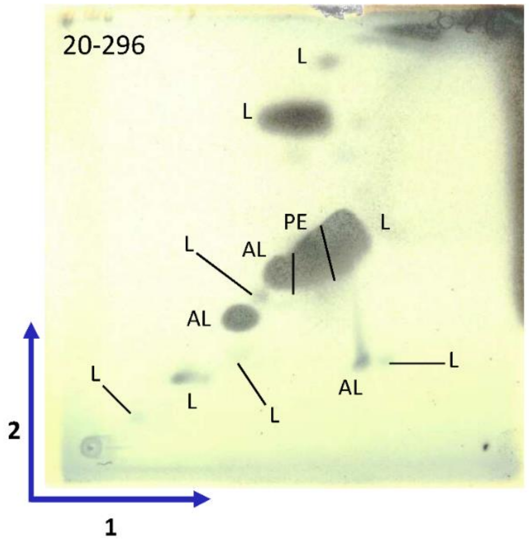

Figure S4: Polar lipid profile of strain APC 3343^T after separation by two-dimensional thin layer chromatography.

PE = Phosphatidylethanolamine; AL= Aminolipid; L = Lipid.

Table S3: Cellular polar lipid profiles of strain APC 3343^T and closely related *Winogradskyella* spp.: 1, APC 3343^T (data from this study); 2, *W. algae* Kr9-9^T (data from [1]); 3, *W. damuponensis* F081-2^T [2]; 4, *W. eximia* KMM 3944^T [3]; 5, *W. litoriviva* KMM 6491^T [4]; 6*, W. endarachnes* HL2-2^T [5].

strain	polar lipid profile
**1**	phosphatidylethanolamine, 3x unknown aminolipids, 8x unknown lipids
**2**	phosphatidylethanolamine, 3x unidentified aminolipids, 1x unidentified lipid
**3**	phosphatidylethanolamine, 1x unidentified aminolipid, 1x unidentified phospholipid, 1x unidentified aminophospholipid, 6x unidentified lipids
**4**	phosphatidylethanolamine, 1x unknown aminolipid
**5**	phosphatidylethanolamine, 2x unknown aminolipids, 2x unknown lipids
**6**	phosphatidylethanolamine, 1x unidentified phospholipid, 2x unidentified aminolipids, 5x unknown polar lipids

Table S4: Antibiotic susceptibility profile of strain 3343^T with corresponding zone diameters (mm). Susceptibility and resistance were determined by no growth and growth, respectively.

Antibiotic	Disc content	Zone diameter (mm)	Result
ampicillin	10 µg	14 ±0.2	S
chloramphenicol	30 µg	18 ±1.6	S
erythromycin	15 µg	28 ±2.4	S
gentamicin	10 µg	0 ±0	R
kanamycin	30 µg	0 ±0	R
lincomycin	15 µg	20 ±3.4	S
neomycin	30 µg	0 ±0	R
novobiocin	5 µg	20 ±3.3	S
oleandomycin	15 µg	28 ±2.6	S
penicillin G	10 U	11 ±0.9	S
polymyxin B	300 U	0 ±0	R
rifampcin	30 µg	37 ±3.4	S
streptomycin	10 µg	0 ±0	R
tetracycline	30 µg	14 ±0.5	S

R = resistant; S = susceptible

References

1. **Kurilenko VV, Romanenko LA, Isaeva MP, Svetashev VI, Mikhailov VV**. Winogradskyella algae sp. nov., a marine bacterium isolated from the brown alga. *Antonie Van Leeuwenhoek* 2019;112(5):731-739.
2. **Lee D-H, Cho SJ, Kim SM, Lee SB**. Winogradskyella damuponensis sp. nov., isolated from seawater. *International Journal of Systematic and Evolutionary Microbiology* 2013;63(1):321-326.
3. **Nedashkovskaya OI, Kukhlevskiy AD, Zhukova NV**. Winogradskyella ulvae sp. nov., an epiphyte of a Pacific seaweed, and emended descriptions of the genus Winogradskyella and Winogradskyella thalassocola, Winogradskyella echinorum, Winogradskyella exilis and Winogradskyella eximia. *International Journal of Systematic and Evolutionary Microbiology* 2012;62(7):1450-1456.
4. **Nedashkovskaya OI, Kukhlevskiy AD, Zhukova NV, Kim SJ, Rhee SK et al.** Winogradskyella litoriviva sp. nov., isolated from coastal seawater. *Int J Syst Evol Microbiol* 2015;65(10):3652-3657.
5. **Xu Y, Li J, Hu Y, Li H, Peng T et al.** Winogradskyella endarachnes sp. nov., a marine bacterium isolated from the brown alga Endarachne binghamiae. *International Journal of Systematic and Evolutionary Microbiology* 2020;71(1).
